# Hypoxia inducible factor 1α-driven steroidogenesis impacts systemic hematopoiesis

**DOI:** 10.1186/s11658-025-00777-9

**Published:** 2025-08-25

**Authors:** Deepika Watts, Nicolas Eberz, Mangesh T. Jaykar, Anupam Sinha, Cagdas Ermis, Johanna Tiebel, Ulrike Baschant, Martina Rauner, Tatyana Grinenko, Triantafyllos Chavakis, Peter Mirtschink, Ali El-Armouche, Ben Wielockx

**Affiliations:** 1 Institute of Clinical Chemistry and Laboratory Medicine, Technische Universität Dresden, 01307 Dresden, Germany; 2 Bone Lab, MK3, Technische Universität Dresden, 01307 Dresden, Germany; 3 Department of Pharmacology and Toxicology, Faculty of Medicine, Technische Universität Dresden, 01307 Dresden, Germany; 4 Experimental Centre, Faculty of Medicine, Technische Universität Dresden, 01307 Dresden, Germany; 5 Present Address: Shanghai Institute of Hematology, State Key Laboratory of Medical Genomics, National Research Center for Translational Medicine at Shanghai, Ruijin Hospital Affiliated to Jiao Tong University School of Medicine, Shanghai, China

**Keywords:** Hypoxia inducible factor-1, Glucocorticoid signaling, Hematopoietic stem cells, Chronic stress hematopoiesis, B cells

## Abstract

**Background:**

Glucocorticoids (GCs) are key regulators of hematopoiesis, but the effects of chronically elevated endogenous GC levels on hematopoietic stem cell (HSC) function and immune cell development remain poorly understood.

**Methods:**

We used a mouse model with adrenocortical cell-specific deletion of hypoxia-inducible factor-1 alpha (HIF1α; P2H1^Ad.Cortex^), which results in sustained and systemic elevation of GC. Hematopoietic stem and progenitor cell (HSPC) populations were analyzed phenotypically and functionally. Transplantation assays assessed the regenerative capacity of HSCs. To determine the role of glucocorticoid receptor (GR) signaling, bone marrow from GR-deficient or wild-type donors was transplanted into P2H1^Ad.Cortex^ or wild-type (WT) recipients.

**Results:**

Chronic GC exposure in P2H1^Ad.Cortex^ mice resulted in HSPC expansion and promoted HSC quiescence and metabolic restraint. Functionally, these HSCs showed enhanced regenerative capacity with superior donor chimerism upon transplantation. There was a marked increase in myeloid progenitors and their progeny, including monocytes and granulocytes. In contrast, B-cell development was significantly impaired, with a developmental block at the pre-pro-B-cell stage. Transplantation studies confirmed that these effects were dependent on GR signaling.

**Conclusions:**

Our study reveals a critical role for chronic GC–GR signaling in modulating HSC function, promoting myeloid output, and impairing B-cell development. The P2H1^Ad.Cortex^ mouse model provides a valuable system to study the hematopoietic consequences of prolonged endogenous glucocorticoid exposure and may aid in understanding the hematologic complications of chronic GC therapy.

**Supplementary Information:**

The online version contains supplementary material available at 10.1186/s11658-025-00777-9.

## Introduction

Hematopoiesis, the process responsible for generating blood cells, is fundamental to the biological system of all vertebrates. It depends primarily on hematopoietic stem cells (HSCs), a rare and specialized population of cells in the bone marrow, where they maintain lifelong blood production through self-renewal and differentiation into all blood and immune lineages [1, 2]. HSCs are rare and reside in specialized bone marrow niches where the oxygen tension is lower than in many other tissues, but still reflects a physiological oxygen environment adapted to the needs of hematopoietic cells [3, 4]. Beyond reduced oxygen conditions, the bone marrow microenvironment, which includes endothelial cells, perivascular cells, and mesenchymal stem cells, plays a crucial role in regulating HSC maintenance and function [5, 6]. Through continuous interactions with these niche components and systemic signals, hematopoiesis remains highly dynamic, adapting to physiological demands and external stimuli [7]. External factors such as stress, inflammation, infection, and aging further modulate this process, often driving stress-induced hematopoiesis [7–10]. However, the long-term effects of chronic physiological stress on hematopoietic function remain incompletely understood.

The adrenal gland plays a critical role in stress adaptation by regulating steroidogenesis, which affects immune function and systemic homeostasis [11, 12]. Synthetic glucocorticoids—often referred to as corticosteroids—are widely used in immunosuppressive therapies, particularly in inflammatory and autoimmune diseases [13]. Our previous findings showed that modulation of hypoxia-inducible factor-1 alpha (HIF1α) expression/activity in the adrenal cortex dramatically alters adrenal steroidogenesis, leading to systemic changes in circulating glucocorticoids and inflammatory cytokines [14]. This raises the important question of how chronic increases in endogenous glucocorticoid production affect HSC and immune cell development, especially when compared with the well-documented effects of synthetic glucocorticoids, such as dexamethasone, when administered therapeutically [15–18].

Furthermore, with the increasing interest in HIF stabilizers as potential therapeutic agents, understanding their effects on adrenal steroidogenesis and hematopoiesis is crucial [19]. Our mouse model with HIF1α deficiency in adrenocortical cells (Akr1b7:cre-*PHD2*/*HIF1*^ff/ff^ [P2H1^Ad.cortex^]) [14], accompanied by higher glucocorticoid levels, provides a unique tool to mimic chronic stress, allowing us to evaluate the effects of elevated endogenous glucocorticoid levels on hematopoietic function. This model also provides insights into the potential adverse effects of HIF inhibitors on adrenal steroidogenesis and immune regulation.

In this study, we show that HIF1α deficiency in the adrenal cortex leads to systemic glucocorticoid overproduction, which promotes HSPC expansion while enforcing a more quiescent HSC state at steady state. These HSCs exhibit a competitive advantage in repopulating the bone marrow after irradiation. In addition, we reveal the complex effects of chronic enhanced glucocorticoids on myeloid and lymphoid maturation. Our findings highlight the indispensable role of HIF1α in systemic physiological regulation and position our mouse model as a valuable tool for understanding the pathophysiological consequences of glucocorticoid dysregulation in human health and disease.

## Methods

### Mice

Akr1b7:cre-*Phd2*/*Hif1*^*ff/ff*^ (P2H1^Ad.Cortex^ or P2H1) and Akr1b7:cre-*Phd2*/*Phd3*^*ff/ff*^ (P2P3^Ad.Cortex^ or P2P3) mice were generated as previously reported by us (no unspecific hematopoietic targeting was detected [data not shown]) [14]. C57BL/6 (B6) and B6.SJL-PtprcaPep3b/BoyJ (SJL) mice were crossed to generate F1 progeny (CD45.1/CD45.2) for transplantation experiments. B6.Cg-Nr3c1tm1.1Jda/J (GR^f/f^; C57BL/6 J) mice were purchased from the Jackson Laboratory [20] and crossed to B6.Cg-Tg(VAV1-cre)1Graf/MdfJ (VavCre; C57BL/6 J) mice [21] to produce Vav:cre-*GR*^*f/f*^ (GR^HSC^) mice. All mice were bred as homozygous for GR^f/f^ and heterozygous for Cre. Cre-negative GR^f/f^ mice were used as WT littermates to GR^HSC^. All mice were maintained under specific pathogen-free (SPF) conditions. For each experiment, transgenic mice were compared with littermate controls (Cre-negative). Mice were genotyped using previously described primers [14]. To assess genotype effects under varying physiological conditions, mice of both sexes ranging in age from 16 weeks to 1 year were included. Since absolute hematopoietic cell numbers can vary by age and sex, the data from each experiment were normalized to internal WT controls before being pooled. This normalization minimized unrelated variability and ensured that the observed differences reflected the genotype effect.

### Flow cytometry and single-cell sorting

Single-cell suspensions were prepared from bone/bone marrow, lymph nodes, thymus, and spleen samples by mechanical disruption and filtration through 70 μm cell strainers (Becton Dickinson, San Diego, CA, USA). The cell suspension from bone marrow and spleen samples was subjected to red blood cell lysis with ACK lysis buffer (Life Technologies Cat. A10492-01). The single-cell suspensions were then stained with the appropriate antibodies. Prior to staining, the cells were incubated with the bio mix of biotinylated antibodies for bone marrow (BM) and in the next round stained with fluorophore-conjugated antibodies. All antibodies were either from ThermoFisher Scientific (eBioscience); BioLegend or BD Biosciences (Supplementary Table I). Cell sorting was performed using a BD Aria II or III as described previously [22]. For the bulk RNAseq experiment, HSC samples were sorted from five individual P2H1^Ad.Cortex^ mice and compared with sorted HSC samples from four individual WT littermates (Fig. S1C).

### Transplantation

For HSC transplantation, HSCs (cKit^+^ Sca-1^+^ CD48^–^ CD150^+^) were isolated from the BM of P2H1^Ad.Cortex^ and WT littermates using BD fluorescence-activated cell sorting (Aria II). Cells were competitively transplanted together with 5 × 10^5^ B6.SJL (CD45.1) total BM competitor cells into lethally irradiated (9 Gy) F1 recipients. For the GC–GR transplantation experiments, lethally irradiated recipient P2H1^Ad.Cortex^ and WT littermates were intravenously injected with 5 × 10^6^ erythrolyzed total BM cells from GR^HSC^ or WT. BM, spleen, or peripheral blood were analyzed at different time points as indicated in the text using a BD LSR Fortessa^™^ Cell Analyzer or BD FACSymphony-A5.

### Statistical analysis

To assess statistical significance between two experimental groups, a Mann–Whitney *U*-test was used for non-normally distributed data, while an unpaired *t*-test with Welch’s correction, accounting for unequal variances, was applied for normally distributed data. Normality was tested using the Shapiro–Wilk test. Statistical differences presented in the figures were considered significant at *p*-values below 0.05. All statistical analyses were performed using GraphPad Prism v10.02 for Windows or higher (GraphPad Software, La Jolla, California, USA, www.graphpad.com).

Additional methods may be found in a data supplement available with the online version of this article.

## Results

### HIF1α-dependent chronic glucocorticoid exposure promotes HSPC expansion and enforces HSC quiescence via p53 pathway activation

**Fig. 1:**
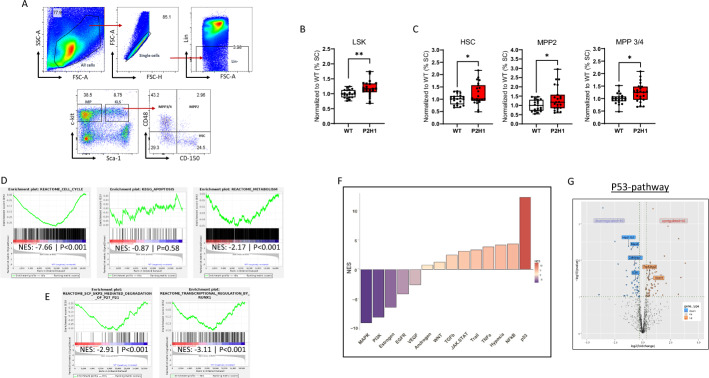
HIF1α-associated steroidogenesis affects hematopoietic stem cell dynamics. **A** FACS gating strategy for identification of HSPC by staining surface markers, as detailed in the Methods section. **B**, **C** Normalized percentage of single cells in the BM of WT mice and Akr1b7:cre-*PHD2*/*HIF1*^ff/ff^ (P2H1) littermates. LSK (Lin^−^ Sca-1^+^ cKit^+^) represents HSPCs. Data points represent individual mice from at least seven different experiments—normalized values against WT control. Data are represented as box and whisker plots showing all data points with whiskers from minimum to maximum. Statistical significance was determined using a Mann–Whitney *U*-test or unpaired *t*-test with Welch’s correction (*p < 0.05; **p < 0.005). **D** GSEA shows significantly decreased cell cycle and metabolism gene signatures and (**E**) differential changes in the regulation of central cell cycle regulators. **F** Carnival analysis displaying pathway activity scores as normalized enrichment score (NES). **G** A volcano plot depicting the genes associated with the p53-pathway, including a selection of up/down regulated genes

Previously, we have shown that chronic inhibition of HIF1α in the adrenal cortex enhances steroidogenesis within the gland, leading to elevated systemic steroid levels, including a sustained and approximately twofold increase in glucocorticoids [14]. Recognizing the pivotal roles of glucocorticoids in immune regulation, stress response, and hematopoiesis, we aimed to investigate the chronic effects of HIF1α-dependent increased steroidogenesis on the hematopoietic system. To this end, we analyzed the bone marrow hematopoietic stem and progenitor cell (HSPC) populations in our P2H1^Ad.Cortex^ mice using fluorescence-activated cell sorting (FACS) and a well-established panel of markers (Fig. 1A). Interestingly, our analyses reveal a slight but significant increase frequency of HSCs (Lin^−^ Kit^+^ Sca-1^+^ CD48^−^ CD150^+^) and multipotent progenitors (MPPs) in P2H1^Ad.Cortex^ mice compared with WT littermate controls. Specifically, we observed an increase in MPP2 (Lin^−^ Kit^+^ Sca-1^+^ CD48^+^ CD150^+^) and MPP3/4 (Lin^−^ Kit^+^ Sca^+^ CD48^+^ CD150^−^), both as a percentage of single cells (Fig. 1B, C) and in total cell numbers (Supplementary Fig. 1A, B). To further explore the behavior of these HSCs, we performed bulk RNA sequencing on sorted HSCs from both WT and P2H1^Ad.Cortex^ mice (Supplementary Fig. 1C). Gene set enrichment analysis (GSEA) revealed a pronounced decrease in cell cycle activity and a dramatic reduction in metabolism in P2H1^Ad.Cortex^ HSCs, with no observed changes in cell survival/apoptosis (Fig. 1D and Supplementary Fig. 1D). Consistent with these findings, we detected a significant reduction in stem cell factor (SCF) ubiquitin ligase complex activity, suggesting reduced degradation of key cell cycle inhibitors p27 and p21. In addition, we found a dramatic reduction in RUNX1-associated HSC differentiation, which strongly suggests increased HSC quiescence (Fig. 1E). This coupled with increased HSC numbers indicates that these cells, while chronically less proliferative, exhibit a preference for self-renewal over differentiation. Interestingly, we also performed cell cycle analysis using FACS but observed no significant differences in the proportions of HSCs in G_0_ or non-G_0_ phases between WT and P2H1^Ad.Cortex^ HSCs (Supplementary Fig. 1E). Although this snapshot of cell cycle status may seem at odds with the RNAseq data, it is very well possible that FACS does not capture the cumulative effects of chronic steroid exposure, which are better reflected in the RNAseq results and the observed increase in HSC numbers in P2H1^Ad.Cortex^ mice. In support of all observations that reflect the chronicity of our phenotype, a carnival analysis of the RNA sequencing data highlighted the strong involvement of the p53 pathway, which is known to maintain genomic integrity and promote cell stability by preventing excessive proliferation (Fig. 1F) [23]. Indeed, within this pathway we identified the modulation of a number of different genes associated with a more quiescent stage of the HSCs. For example, the upregulation of *Trp53bp2* and *Slamf1* (*CD150*) supports the quiescent state of HSCs. Trp53bp2 enhances p53-mediated cell cycle arrest and genomic stability, while Slamf1 (CD150) reinforces dormancy by mediating niche interactions and suppressing activation signals. In parallel, the downregulation of *Hsd11b1*, *Mastl*, *E2f1*, and *Cdk5rap1* contributes to the same outcome, collectively forming a regulatory network that maintains HSC quiescence under chronic glucocorticoid exposure. The suppression of cell cycle regulators Mastl and E2f1 increases quiescence by delaying mitotic entry and inhibiting the G1/S transition. Downregulation of Cdk5rap1 reduces mitochondrial activity, contributing to a low-energy state, while Hsd11b1 downregulation, likely due to chronically elevated systemic glucocorticoid levels, may limit intracellular GC activation and prevent HSC hyperactivation (Fig. 1G). Taken together, these combined changes may provide a regulatory network to maintain HSC quiescence under chronic glucocorticoid exposure in P2H1^Ad.Cortex^ mice compared with their WT littermates.

### HSC transplantation reveals enhanced regenerative potential of quiescent P2H1^Ad.Cortex^ HSCs

**Fig. 2:**
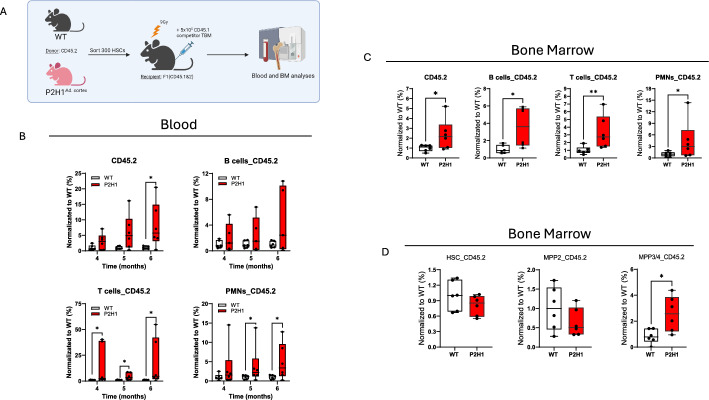
Enhanced replenishment of the hematopoietic system with transplanted hematopoietic stem cells from P2H1 mice. **A** Schematic overview of the HSC-transplantation in irradiated recipients and subsequent blood analyses from 4 months after transplantation and an additional BM analysis at 6 months. **B** Blood analysis and (**C**, **D**) BM FACS analysis of mature hematopoietic populations and HSPCs. Data points represent individual mice from at least two different experiments—normalized values against the average of WT controls. Data are presented as box and whisker plots showing all data points with whiskers from minimum to maximum. Statistical significance was determined using a Mann–Whitney *U*-test or unpaired *t*-test with Welch’s correction (*p < 0.05; **p < 0.005)

To validate these findings, we analyzed the remaining HSC function from both genotypes by transplanting these stem cells into irradiated recipient mice to assess their functional capacity to repopulate the bone marrow. We transplanted 300 donor HSCs (CD45.2) together with 5 × 10^5^ competitive bone marrow (BM) cells (CD45.1) into irradiated WT (F1) recipient mice (CD45.1 and CD45.2). Donor-derived white blood cell (WBC) chimerism was then monitored monthly starting 4 months post-transplantation (Fig. 2A). Notably, P2H1^Ad.Cortex^-derived HSCs showed a marked increase in chimerism, particularly in circulating PMNs and T lymphocytes, compared with WT controls (Fig. 2B). At 6 months post-transplant, bone marrow analysis of recipient mice revealed a significant increase in P2H1^Ad.Cortex^-derived CD45^+^ mature cells, consistent with the changes observed in the circulation (Fig. 2C). In addition, analysis of HSPC fractions in the BM revealed higher levels of MPP3/4 populations, with a slight decrease in both HSCs and MPP2 populations (Fig. 2D). Taken together, these results support our RNA sequencing data and suggest that the more quiescent P2H1^Ad.Cortex^ HSCs experience less exhaustion and, as a result, have a greater regenerative potential to replenish irradiated bone marrow compared with WT HSCs.

### Increased erythropoiesis independent of bone marrow hematopoiesis

**Fig. 3:**
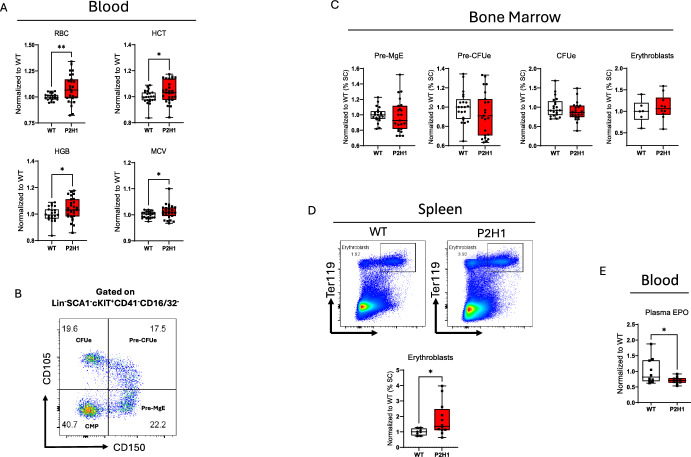
Chronic exposure to glucocorticoids modulates erythropoiesis in P2H1 mice. **A** Normalized number of different RBC parameters in circulation from WT mice and P2H1 littermates under steady state. **B** Representative FACS gating strategy for the identification of erythroid progenitors by staining surface markers, as detailed in the Methods section. **C** Normalized percentage of single cells representing different erythroid progenitors in the BM. **D** Representative FACS gating strategy of erythroblasts in the spleen and significantly increased spleen EB single cell percentages in P2H1 mice. **E** EPO ELISA with plasma samples from WT and P2H1 littermates demonstrating a significant decrease of EPO levels in circulation. Data points represent individual mice from at least two different experiments—normalized values against the average of WT controls. Data are presented as box and whisker plots showing all data points with whiskers from minimum to maximum. Statistical significance was determined using a Mann–Whitney *U*-test or unpaired *t*-test with Welch’s correction (*p < 0.05; **p < 0.005)

Recent studies have shown that chronic exposure to excess glucocorticoids can lead to overproduction of red blood cells (RBCs) in patients with Cushing’s disease [24]. Supported by these findings, we examined blood cells in our P2H1^Ad.Cortex^ mouse model and found similar changes. Specifically, our data show that mice chronically exposed to glucocorticoids exhibit a significant increase in RBC numbers, hemoglobin levels, and their mean corpuscular volume (MCV) (Fig. 3A). We further investigated whether this increase was due to a biased shift towards erythroid progenitors. Analyses of these fractions in the BM (Fig. 3B) revealed no significant differences in the populations of bipotent pre-megakaryocyte erythroid progenitors (Pre-MgE), upstream of the more committed erythroid-restricted progenitors (Pre-CFUe) and erythroid colony-forming units (CFUe) (Fig. 3C). Similarly, erythroblasts (EBs), the immediate precursors of RBCs in the bone marrow, showed no significant variation in P2H1^Ad.Cortex^ mice compared with WT littermate controls (Fig. 3C). Conversely, and consistent with findings from another erythrocytotic mouse model previously studied by our team [25], it is the spleen of P2H1^Ad.Cortex^ mice that engages in stress erythropoiesis, containing significantly more erythroblasts than their WT counterparts (Fig. 3D). In contrast to the previous study in which erythrocytosis was directly induced by increased erythropoietin (EPO) production [25], our P2H1^Ad.Cortex^ mice exhibited significantly lower EPO levels in circulation. This decrease might underlie a negative feedback mechanism possibly induced by the increased number of circulating erythrocytes and the greater potential to deliver oxygen to EPO-producing cells in the kidney, automatically leading to lower HIF2α activity and consequent *Epo* transcription (Fig. 3E). These findings strongly suggest that the spleen engages in mild stress erythropoiesis following long-term exposure to chronically elevated glucocorticoids and corroborates the increased number of splenic erythroblasts observed in P2H1^Ad.Cortex^ mice.

### HIF-mediated chronic increase in steroidogenesis results in modulation of myelopoiesis

Next, we investigated the effects of prolonged chronic exposure to elevated glucocorticoids on myelopoiesis. First, we analyzed myeloid precursors in the bone marrow and found a significant reduction in common myeloid progenitors (CMPs), alongside a notable increase in granulocyte-monocyte progenitors (GMPs) (Supplementary Figs. 2A, 3B, and 4A). This increased GMP level correlated with a slight but significant increase in the number of monocytes and granulocytes, although eosinophils in this compartment were markedly reduced (Supplementary Figs. 2B, C, and 4B). Correspondingly, we observed an increase in circulating monocytes and a decrease in splenic eosinophils (Supplementary Fig. 2D, E). The latter is consistent with previous findings that glucocorticoids can directly regulate eosinophil functionality and survival, potentially leading to lower eosinophil counts [26].

### Increased Treg cells in the peripheral lymphoid organs

**Fig. 4:**
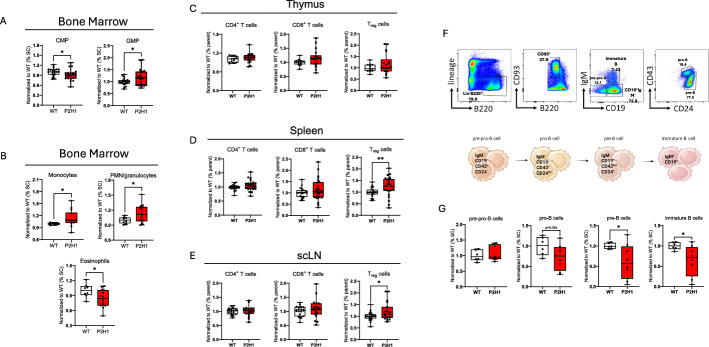
Chronic increased steroidogenesis results in alterations in myeloid cells and lymphocytes. **A**, **B** FACS analysis of BM-derived myeloid progenitors and mature populations (normalized from the % of single cells). **C**–**E** FACS analysis of different T-cell populations in distinct organs (normalized from the % of parent). Each graph represents data from at least three independent experiments. **F** Representative FACS plots of four different B-cell maturation stages and (**G**) FACS analysis of BM-derived B-cell progenitors (normalized from the % of single cells). Data points represent individual mice from at least two different experiments—normalized values against WT control. Data are presented as box and whisker plots showing all data points with whiskers from minimum to maximum. Statistical significance was determined using a Mann–Whitney *U*-test or unpaired *t*-test with Welch’s correction (*p < 0.05; **p < 0.005)

Previous studies have demonstrated that glucocorticoids significantly affect the function and proliferation of regulatory T cells (Tregs), highlighting their importance in the management and treatment of various immune-related diseases [27]. Building on this, we extended our study to assess the impact of HIF1α-mediated chronic glucocorticoid elevation by analyzing different T-cell lineages in both the thymus and peripheral organs, with a specific focus on CD4^+^ and CD8^+^ T cells, and Tregs, identified as CD4^+^ CD25^+^ Foxp3^+^, in particular (Supplementary Fig. 2F). Interestingly, while no differences in CD4^+^ or CD8^+^ T-cell fractions were observed between the organs we examined, Tregs were significantly increased in the spleen and the subcutaneous lymph nodes (scLN) (Fig. 4C–E). Given this increase in Tregs, we assessed the apoptotic activity of Treg cells in the spleen but found no significant differences in viability between P2H1^Ad.Cortex^ Treg cells and their WT counterparts (Supplementary Fig. 2G, H). Therefore, this observation suggests that the increase in Tregs may be primarily due to enhanced peripheral differentiation.

### Chronically elevated glucocorticoids reduce B-cell lymphopoiesis

Previously, glucocorticoids have been shown to directly regulate B-cell maturation in both the bone marrow and peripheral immune compartments [28]. In addition, ischemic-stroke-induced glucocorticoid signaling has been reported to influence hematopoietic B-cell lineage decisions, leading to impaired adaptive immunity [29]. Given these established effects, we investigated whether chronic glucocorticoid overproduction in our P2H1^Ad.Cortex^ mice similarly affects B-cell development. Consistent with previous findings, our analysis revealed significant disruptions in B-cell maturation beyond the pre-pro-B-cell progenitor stage (Lin^−^ B220^int^ CD93^+^ IgM^−^ CD43^+^ CD24^−^) (Fig. 4F). Specifically, we observed a marked reduction in both the number and frequency of pro-B cells (Lin^−^ B220^int^ CD93^+^ IgM^−^ CD19^+^ CD43^+^ CD24^int^), pre-B cells (Lin^−^ B220^int^ CD93^+^ IgM^−^ CD19^+^ CD43^low^ CD24^+^), and immature B cells (Lin^−^ B220^int^ CD93^+^ IgM^+^ CD19^+^) in the bone marrow (Fig. 4G). These findings suggest that the immunosuppressive effects of chronic levels of glucocorticoids extend to early B-cell differentiation and may contribute to B-cell developmental obstruction.

### The GC–GR (GC-receptor) axis regulates myeloid and B-cell differentiation in P2H1^Ad.Cortex^ mice

**Fig. 5:**
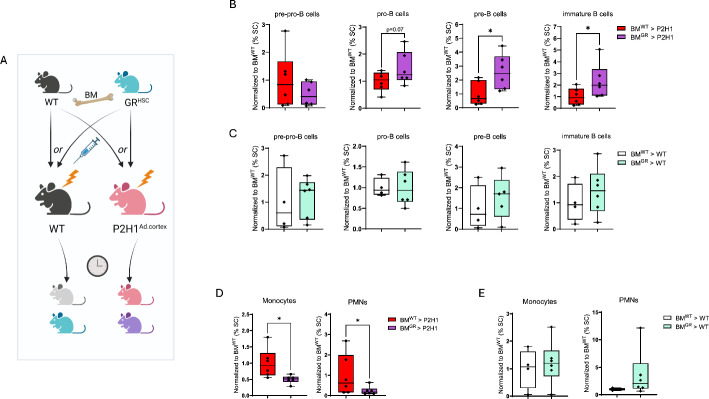
The GC–GR axis regulates myeloid and B-cell differentiation in P2H1^Ad.Cortex^ mice. **A** Schematic overview of the GR^HSC^ or WT BM-transplantation in irradiated P2H1^Ad.Cortex^ or WT recipients. **B**, **C** Subsequent FACS analysis of different B-cell progenitors in the spleen 12 months after transplantation. **D**, **E** FACS analysis of monocytes and PMNs. Data points represent individual mice from two different experiments—normalized values against WT control. Data are presented as box and whisker plots showing all data points with whiskers from minimum to maximum. Statistical significance was determined using a Mann–Whitney *U*-test or unpaired *t*-test with Welch’s correction (*p < 0.05)

To directly confirm that the hematopoietic changes observed in P2H1^Ad.Cortex^ mice are driven by glucocorticoid signaling through the GR (GC–GR axis), we performed transplantation experiments using hematopoietic cells deficient in GR (from Vav:cre-GR^f/f^ [GR^HSC^] mice) or WT controls. These cells were transplanted into P2H1^Ad.Cortex^ or littermate controls, as shown in Fig. 5A. Twelve months post-transplantation, analysis of B-cell fractions in the spleen revealed that the block in pro-, pre-, and immature B-cell differentiation in P2H1^Ad.Cortex^ mice (Fig. 4G) was entirely dependent on GR expression in hematopoietic cells (Fig. 5B); an effect that was absent in their transplanted littermate controls (Fig. 5C). Similarly, P2H1^Ad.Cortex^ mice receiving GR-deficient BM exhibited a significant reduction in monocytes and PMNs, strongly suggesting that myeloid expansion in this model is also directly regulated by GC–GR signaling (Fig. 5D, E). These findings further underscore the significant impact that chronic glucocorticoid overproduction due to HIF1α loss in the adrenal cortex can have on the maturation process of B lymphocytes and myeloid cells.

### HIF1α-induced downregulation of steroidogenesis affects erythropoiesis but not HSPCs

**Fig. 6:**
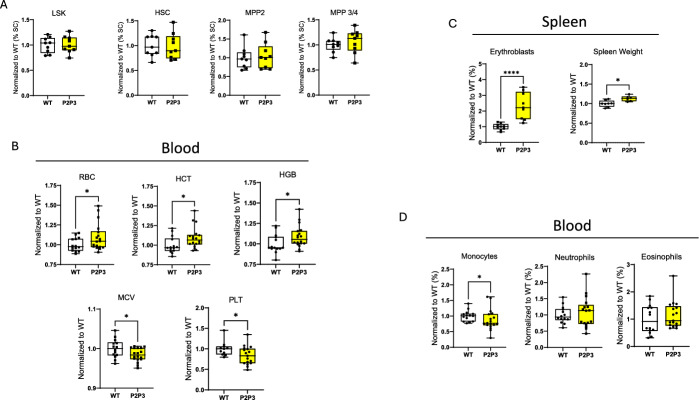
HIF1α-induced downregulation of steroidogenesis affects erythropoiesis but not HSPCs. **A** Normalized percentage of single cells in the BM of WT mice and Akr1b7:cre-PHD2/PHD3^ff/ff^ (P2P3) littermates. Data points represent individual mice from at least three different experiments—normalized values against WT control. Data are represented as means ± SEM. **B** Normalized number of different RBC parameters in circulation from WT mice and P2P3 littermates under steady state. **C** Representative FACS gating strategy for the identification of erythroblasts and weight of the representative spleen. **D** Sysmex analysis of myeloid cells in the blood (normalized to the average of WT controls). Data points represent individual mice from two different experiments—normalized values against WT control. Data are presented as box and whisker plots showing all data points with whiskers from minimum to maximum. Statistical significance was determined using a Mann–Whitney *U*-test or unpaired *t*-test with Welch’s correction (*p < 0.05)

In our previous study, we demonstrated that simultaneous deficiency of PHD2 and PHD3 in the adrenal cortex leads to increased HIF1α activity, resulting in a sustained reduction in steroidogenesis, including lower glucocorticoid levels in both the adrenal gland and the circulation [14]. To further investigate the hematopoietic effects of this condition, we analyzed hematopoietic cells in the P2P3^Ad.Cortex^ mouse line (P2P3), using the same approach as for P2H1^Ad.Cortex^ mice. While elevated glucocorticoids significantly increased HSPC fractions in P2H1^Ad.Cortex^ mice (Fig. 1B), we found no significant changes in hematopoietic stem and progenitor cells (HSPCs) in P2P3^Ad.Cortex^ mice compared with WT littermates (Fig. 6A and Supplementary Fig. 3A). However, further analysis revealed an increase in RBC levels, accompanied by higher hematocrit (HCT) and hemoglobin (HGB) levels, but lower mean corpuscular volume (MCV) and platelet counts (Fig. 6B). Consistent with these findings, secondary erythropoiesis was enhanced in the spleen rather than the bone marrow, correlating with increased spleen weight (Fig. 6C and Supplementary Fig. 3B). Analysis of white blood cells (WBCs) and their precursors showed a significant reduction in circulating monocytes, whereas other myeloid cells, T cells, and B cells remained unchanged (Fig. 6D and Supplementary Fig. 3C, D). Taken together, these results indicate that P2P3^Ad.Cortex^ mice, despite chronically producing lower levels of glucocorticoids, do not exhibit an inverse phenotype of P2H1^Ad.Cortex^ mice. This underscores the complex interplay between reduced glucocorticoid levels and hematopoiesis, emphasizing that changes in steroidogenesis do not necessarily translate into direct or linear effects on HSPCs and lineage differentiation.

## Discussion

Our study demonstrates that chronic glucocorticoid exposure, driven by HIF1α loss in adrenocortical cells in vivo, expands HSPCs while shifting HSCs toward a more quiescent and metabolically restrained state. Functionally, these HSCs exhibited enhanced regenerative potential, while myeloid differentiation was significantly altered, with an increase in GMPs, monocytes, and PMNs, but a dramatic inhibition of B-cell differentiation. In a transplantation assay using GR-deficient bone marrow into irradiated P2H1^Ad.Cortex^ mice, we confirmed that the myeloid and B-cell differentiation phenotypes were driven by glucocorticoid receptor (GC–GR) signaling. In contrast, HIF1α-induced downregulation of steroidogenesis in P2P3^Ad.Cortex^ mice had no significant effect on HSPC numbers, B-cell differentiation, or Treg profiles. Despite lower systemic glucocorticoids, these mice did not exhibit the inverse phenotype of P2H1^Ad.Cortex^ mice, highlighting the complex interplay between steroid hormones and hematopoiesis.

Previous studies on the role of glucocorticoids in HSCs primarily focused on short-term dexamethasone treatment, a synthetic glucocorticoid, which differs from our chronic endogenous approach [16, 30, 31]. However, consistent with previous experiments using only low doses of dexamethasone [17] our analysis revealed increased HSC quiescence, leading to a significant delay in HSC exhaustion, as observed in our long-term transplantation experiment. At the molecular level, this prolonged quiescent state appears to be influenced by key regulators that we identified through our RNAseq analysis, which suggest a coordinated network reinforcing chronically mild cell cycle arrest and niche interactions to preserve HSC function under chronic glucocorticoid exposure (p53 pathway activity). Upregulation of *Trp53bp2* likely enhances HSC quiescence through complementary mechanisms. As an enhancer of p53 signaling, Trp53bp2 promotes cell cycle arrest and maintains genomic stability [32], potentially preventing HSCs from entering an active proliferative state. This is consistent with the broader role of p53 in enforcing quiescence under conditions of cellular stress, including in HSCs [33]. In parallel, Slamf1 (CD150) is a critical marker of quiescent HSCs, particularly in the long-term stem cell compartment. It is involved in maintaining HSC quiescence by mediating interactions between HSCs and their niche, promoting signals that support dormancy, and may therefore improve survival after transplantation [34]. The downregulation of *Hsd11b1*, *Mastl*, *E2f1*, and *Cdk5rap1* in HSCs adds up to the coordinated shift toward quiescence. Mastl and E2f1 are both key regulators of the cell cycle, with their suppression leading to inhibition of proliferation—Mastl downregulation increases PP2A-B55, a phosphatase complex that dephosphorylates mitotic substrates, thereby delaying mitotic entry [35], while E2f1 suppression reduces the expression of genes required for G1/S transition [36]. In parallel, Cdk5rap1 downregulation limits mitochondrial activity, promoting a low-energy state that supports dormancy [37]. Uniquely, Hsd11b1 downregulation in our model may result from chronically elevated systemic glucocorticoid levels, serving as a protective mechanism to limit intracellular glucocorticoid activation and potentially preventing HSC activation [38]. Together, these molecular changes likely work synergistically to stabilize HSC quiescence in response to chronic glucocorticoid exposure and preserve the long-term integrity of the stem cell pool.

Our P2H1^Ad.Cortex^ mice exhibited marked alterations in mature hematopoietic cells, particularly enhanced erythropoiesis. This mirrors observations in patients with Cushing’s disease, where chronic glucocorticoid exposure is linked to erythrocytosis, elevated hematocrit, and increased hemoglobin levels. Consistent with this, our analysis revealed a significant increase in RBC numbers, hemoglobin levels, and MCV in P2H1^Ad.Cortex^ mice, despite reduced EPO levels. These findings suggest that glucocorticoids directly promote erythropoiesis, operating through an EPO-independent mechanism. Our findings are consistent with earlier work demonstrating that glucocorticoids act synergistically with erythropoietin to promote the expansion and differentiation of erythroid progenitors [39]. Moreover, this is supported by previous studies indicating that glucocorticoids can directly enhance stress erythropoiesis, particularly in the spleen, which aligns with our observation of increased splenic erythroblast populations [24, 40]. Interestingly, our P2P3^Ad.Cortex^ mice, characterized by downregulated glucocorticoid production, also exhibited a significant increase in RBC counts, although the MCV was significantly reduced in P2P3^Ad.Cortex^ mice compared with wild-type controls. This observation contrasts with typical clinical findings, as conditions of glucocorticoid deficiency, such as in Addison’s disease, are generally associated with anemia rather than erythrocytosis [41]. Therefore, the mechanisms underlying the increased RBC production in our P2P3^Ad.Cortex^ mice remain unclear and warrant further investigation to elucidate the background of this observed phenotype.

We also observed alterations in myeloid cell populations within the bone marrow and, to a lesser extent, in the spleen. Conversely, eosinophil counts were significantly reduced in these compartments. These findings align with clinical observations in Cushing’s disease, where chronic glucocorticoid excess is associated with elevated myeloid cells, while eosinophil levels are typically decreased, due to suppression of their production [24]. Furthermore, we observed significant alterations in lymphocyte populations, most notably a severe impairment in B-cell development within the bone marrow. Specifically, pro-B, pre-B, and immature B cell populations were significantly reduced, indicating a blockade in B-cell maturation beyond the pre-pro-B-cell stage. This finding is consistent with reports that glucocorticoids suppress B lymphopoiesis, either through direct effects on B-cell progenitors or by modifying the bone marrow microenvironment [29, 42].

Finally, our transplantation experiments provide decisive evidence that the observed increase in monocytes and PMNs, and the inhibition in B-cell maturation, are directly mediated by glucocorticoid receptor signaling. The failure of B-cell differentiation beyond the pro-pre-B-cell stage was entirely dependent on the presence of GR in hematopoietic cells, as this phenotype was absent when GR-deficient HSCs were transplanted in P2H1^Ad.Cortex^ mice. Similarly, mice receiving GR-deficient BM exhibited significantly lower monocyte and PMN numbers, confirming that the myeloid expansion observed in P2H1^Ad.Cortex^ mice is also related to the GR. These findings establish a clear GC–GR-dependent axis regulating both myeloid and B-cell lineage commitment, further underscoring the profound impact of low-dose, chronic glucocorticoid overproduction on the hematopoietic system.

A potential limitation of this study is that systemic glucocorticoid and adrenocorticotropic hormone (ACTH) levels were not remeasured in the cohorts analyzed for hematopoiesis. However, these mouse lines have been extensively validated for their endocrine profiles in previous studies [14]. We continuously confirmed genotype integrity and monitored blood parameters to ensure consistency with these data. In addition, although serial transplantation is considered the most rigorous method for evaluating long-term HSC self-renewal, we used competitive transplantation assays, a well-established and widely accepted technique, for identifying functional differences in repopulation capacity.

An important next step is to determine if the hematopoietic alterations observed in our model can be reproduced by administering exogenous glucocorticoids. This comparison will clarify if these phenotypes primarily result from chronic, physiologically regulated increases in endogenous steroid production or if they can also occur under therapeutic corticosteroid regimens. Answering this question is critical to assessing the translational relevance of our findings and the potential of this model to predict steroid-related hematologic complications.

Taken together, our findings reveal that chronic glucocorticoid overproduction profoundly alters hematopoiesis, promoting HSPC expansion, increasing quiescence, and enhancing the regenerative capacity of HSCs, while also shaping lineage differentiation. We demonstrate that GC–GR signaling is a key regulator of myelopoiesis and B lymphopoiesis, driving an increase in monocytes and PMNs, while blocking B-cell maturation in the bone marrow. Decisively, our transplantation experiments confirm that these effects are intrinsic to hematopoietic cells and directly mediated by GR signaling. Therefore, this adrenocortical-specific HIF1α-deficient mouse model provides a powerful tool for further investigating how chronic glucocorticoid exposure impacts HSC function, immune cell differentiation, and stress hematopoiesis. By dissecting the precise molecular mechanisms underlying these changes, this system may offer valuable insights into glucocorticoid-related hematological disorders and immunosuppressive therapies, ultimately contributing to a better understanding of how HIF-dependent steroid hormone imbalances influence hematopoiesis in health and disease.

## Supplementary Information

Additional file 1.

Additional file 2.

Additional file 3.

## Data Availability

RNAseq data are available at GEO (GSE292877) [NCBI tracking system no. 25087899]. Data are available from the authors upon reasonable request and with permission of (Ben.Wielockx@tu-dresden.de).

## Abbreviations

ACK: Ammonium–chloride–potassium
APL: Acute promyelocytic leukemia
AML: Acute myelocytic leukemia
BM: Bone marrow
CFUe: Colony-forming unit erythroid
CMP: Common myeloid progenitor
Cre: Cre recombinase
EB: Erythroblast
EPO: Erythropoietin
FACS: Fluorescence-activated cell sorting
GMP: Granulocyte-monocyte progenitor
GR: Glucocorticoid receptor
GRHSC: Glucocorticoid receptor-deficient hematopoietic stem cells
GSEA: Gene set enrichment analysis
HCT: Hematocrit
HGB: Hemoglobin
HIF: Hypoxia-inducible factor
HIF1α: Hypoxia-inducible factor-1 alpha
HSC: Hematopoietic stem cell
HSPC: Hematopoietic stem and progenitor cell
LSK: Lin^−^ Sca-1^+^ cKit^+^ (marker combination for HSPCs)
MCV: Mean corpuscular volume
MPP: Multipotent progenitor
P2H1: Akr1b7:cre-PHD2/HIF1^ff/ff^ mice
P2P3: Akr1b7:cre-PHD2/PHD3^ff/ff^ mice
PMN: Polymorphonuclear neutrophil
Pre-CFUe: Pre-colony-forming unit erythroid
Pre-MgE: Pre-megakaryocyte erythroid progenitor
RBC: Red blood cell
RNAseq: RNA sequencing
SCF: Stem cell factor
SPF: Specific pathogen-free
Treg: Regulatory T cell
WT: Wild-type
