# Supplementary material for: Hypoxia inducible factor 1α-driven steroidogenesis impacts systemic hematopoiesis
Source: Cell Mol Biol Lett. 2025 Aug 25;30:101. doi: 10.1186/s11658-025-00777-9 (PMC12379443; doi:10.1186/s11658-025-00777-9)
Supplement: Supplementary file 1 — Additional file 1. [file 11658_2025_777_MOESM1_ESM.docx]

**Supplementary Methods**

# **Blood analysis**

Peripheral blood was drawn from mice by retro-orbital sinus puncture using heparinized micro hematocrit capillaries (VWR, Darmstadt, Germany) and blood was further diluted with PBS (1:5) according to the user manual. White blood cells, red blood cells and platelets were measured in whole blood using a Sysmex automated blood cell counter (Sysmex 117 XE-5000), and plasma was separated and stored at -80 °C.

# **Apoptosis and Cell cycle**

For apoptosis analysis, cells were first surface-stained, washed twice with 200 µl cold PBS, and then resuspended in 1ml 1X Binding Buffer. Annexin V (556420) (BD-Pharmingen) was then added to 100ul of suspension and cells were incubated for 20 minutes in the dark at room temperature, followed by DAPI staining. For analysis, cells that were negative for DAPI and Annexin V were considered to be alive, while cells that were positive for DAPI and Annexin V were considered to be dead. Furthermore, Annexin V-positive, DAPI-negative cells were considered to be undergoing apoptosis. Cell Cycle analysis was performed as described previously [1]. Briefly, HSCs were fixed, permeabilized and stained for intracellular Ki-67 (PE, B56, BD Biosciences) to differentiate between G0 and G1 phases. DAPI was used to measure DNA content and to separate cells in S/G2/M phase from G0 and G1 cells.

# **Transcriptome Mapping**

Low quality nucleotides were removed using Illumina fastq filter (<http://cancan.cshl.edu/labmembers/gordon/fastq_illumina_filter/>). Reads were further subjected to adaptor trimming using cutadapt [2]. Alignment of the reads to the Mouse genome was done using STAR Aligner [3] using the parameters: “--runMode alignReads --outSAMstrandField intronMotif --outSAMtype BAM SortedByCoordinate --readFilesCommand zcat”. Mouse Genome version GRCm38 (release M12 GENCODE) was used for the alignment.

# **Read Quantification**

Using the parameters: 'htseq-count -f bam -s reverse -m union -a 20', HTSeq-0.6.1p1 [4] was used to count the reads that map to the genes in the aligned sample files. The GTF file (gencode.vM12.annotation.gtf) used for read quantification was downloaded from Gencode (https://www.gencodegenes.org/mouse/release_M12.html).

# **Differential Expression Analysis**

Gene centric differential expression analysis was performed using DESeq2_1.8.1 [5]. Also, the raw read counts for the genes across the samples were normalized using 'rlog' command of DESeq2 and subsequently these values were used to render a PCA plot using ggplot2_1.0.1 [6]. Heatmaps were generated using Complex Heatmap package of R/Bioconductor [7].

# **Functional Analyses**

Pathway and functional analyses was performed using GSEA[8] and EGSEA [9]. GSEA/EGSEA were run using normalized gene expression matrix against databases like Molecular Signatures Database (MSigDB), Reactome, KEGG and GO based repositories.

# **Pathway Activity**

Additionally, the pathway activity calculations were performed using progeny [10]. A R package progeny is a compendium of 14 pathways, which have been extracted from publicly available perturbation experiments. The input to the progeny() command was the normalized gene expression matrix.

# **References**

1. Singh RP, Franke K, Kalucka J, Mamlouk S, Muschter A, Gembarska A, et al. HIF prolyl hydroxylase 2 (PHD2) is a critical regulator of hematopoietic stem cell maintenance during steady-state and stress. Blood. 2013;121(26):5158-66. doi: 10.1182/blood-2012-12-471185.

2. Martin M. Cutadapt removes adapter sequences from high-throughput sequencing reads. 2011. 2011;17(1):3. doi: 10.14806/ej.17.1.200.

3. Dobin A, Davis CA, Schlesinger F, Drenkow J, Zaleski C, Jha S, et al. STAR: ultrafast universal RNA-seq aligner. Bioinformatics. 2012;29(1):15-21. doi: 10.1093/bioinformatics/bts635.

4. Anders S, Huber W. Differential expression analysis for sequence count data. Genome Biology. 2010;11(10):R106. doi: 10.1186/gb-2010-11-10-r106.

5. Anders S, Pyl PT, Huber W. HTSeq—a Python framework to work with high-throughput sequencing data. Bioinformatics. 2014;31(2):166-9. doi: 10.1093/bioinformatics/btu638.

6. Wickham H. ggplot2: elegant graphics for data analysis. USE R. New York Springer-Verlag; 2016.

7. Gu Z, Eils R, Schlesner M. Complex heatmaps reveal patterns and correlations in multidimensional genomic data. Bioinformatics. 2016;32(18):2847-9. doi: 10.1093/bioinformatics/btw313.

8. Subramanian A, Tamayo P, Mootha VK, Mukherjee S, Ebert BL, Gillette MA, et al. Gene set enrichment analysis: a knowledge-based approach for interpreting genome-wide expression profiles. Proc Natl Acad Sci U S A. 2005;102(43):15545-50. doi: 10.1073/pnas.0506580102.

9. Alhamdoosh M, Ng M, Wilson NJ, Sheridan JM, Huynh H, Wilson MJ, et al. Combining multiple tools outperforms individual methods in gene set enrichment analyses. Bioinformatics. 2017;33(3):414-24. doi: 10.1093/bioinformatics/btw623.

10. Schubert M, Klinger B, Klünemann M, Sieber A, Uhlitz F, Sauer S, et al. Perturbation-response genes reveal signaling footprints in cancer gene expression. Nature Communications. 2018;9(1):20. doi: 10.1038/s41467-017-02391-6.
