# Supplementary material for: Hypoxia inducible factor 1α-driven steroidogenesis impacts systemic hematopoiesis
Source: Cell Mol Biol Lett. 2025 Aug 25;30:101. doi: 10.1186/s11658-025-00777-9 (PMC12379443; doi:10.1186/s11658-025-00777-9)
Supplement: Supplementary file 2 — Additional file 2. [file 11658_2025_777_MOESM2_ESM.docx]

**Supplementary Table 1: Overview of the antibodies used in the different experiments**

| **Antibodies** | **Host** | **Cat. Number** | **Company** |
| --- | --- | --- | --- |
| CD3e Monoclonal Antibody (145-2C11), Biotin, eBioscience™ (1:1000) | Armenian Hamster | 13-0031-82 | Invitrogen |
| CD19 Monoclonal Antibody (eBio1D3 (1D3)), Biotin, eBioscience™ (1:500) | Rat | 13-0193-81 | Invitrogen |
| NK1.1 Monoclonal Antibody (PK136), Biotin, eBioscience™ (1:2000) | Mouse | 13-5941-81 | Invitrogen |
| TER-119 Monoclonal Antibody (TER-119), Biotin (1:200) | Rat | MA5-17819 | Invitrogen |
| CD11b Monoclonal Antibody (M1/70), Biotin, eBioscience™ (1:500) | Rat | 13-0112-81 | Invitrogen |
| Ly-6G/Ly-6C Monoclonal Antibody (RB6-8C5), Biotin, eBioscience™ (1:800) | Rat | 13-5931-82 | Invitrogen |
| CD45R (B220) Monoclonal Antibody (RA3-6B2), Biotin, eBioscience™ (1:400) | Rat | 13-0452-82 | Invitrogen |
| CD16/CD32 Monoclonal Antibody (93), Alexa Fluor™ 700, eBioscience™ (1:50) | Rat | 56-0161-82 | Invitrogen |
| APC anti-mouse CD48 Antibody (1:300) | Armenian Hamster | 103412 | BioLegend |
| PE/Cyanine7 anti-mouse CD150 (SLAM) Antibody (1:100) | Rat | 115914 | BioLegend |
| CD117 (c-Kit) Monoclonal Antibody (2B8), APC-eFluor™ 780, eBioscience™ (1:600) | Rat | 47-1171-80 | Invitrogen |
| Ly-6A/E (Sca-1) Monoclonal Antibody (D7), PE-Cyanine5, eBioscience™ (1:100) | Rat | 15-5981-82 | Invitrogen |
| CD105 (Endoglin) Monoclonal Antibody (MJ7/18), PE, eBioscience™ (1:400) | Rat | 12-1051-82 | Invitrogen |
| CD34 Monoclonal Antibody (RAM34), FITC, eBioscience™ (1:50) | Rat | 11-0341-85 | Invitrogen |
| CD41a Monoclonal Antibody (eBioMWReg30 (MWReg30)), PerCP-eFluor™ 710, eBioscience™ (1:400) | Rat | 46-0411-82 | Invitrogen |
| eBioscience™ Streptavidin eFluor™ 450 Conjugate (1:300) |  | 48-4317-82 | Invitrogen |
| CD3e Monoclonal Antibody (eBio500A2 (500A2)), Alexa Fluor™ 700, eBioscience™ (1:100) | Armenian Hamster | 56-0033-82 | Invitrogen |
| CD45R (B220) Monoclonal Antibody (RA3-6B2), PE-Cyanine7, eBioscience™ (1:500) | Rat | 25-0452-82 | Invitrogen |
| CD11b Monoclonal Antibody (M1/70), eFluor™ 450, eBioscience™ (1:800) | Rat | 48-0112-80 | Invitrogen |
| Ly-6G Monoclonal Antibody (1A8-Ly6g), APC, eBioscience™(1:200) | Rat | 17-9668-80 | Invitrogen |
| BD Pharmingen™ FITC Rat Anti-Mouse Ly-6C (1:300) | Rat | 553104 | BD |
| TER-119 Monoclonal Antibody (TER-119), PE-Cyanine5, eBioscience™ (1:100) | Rat | 15-5921-83 | Invitrogen |
| F4/80 Monoclonal Antibody (BM8), PE, eBioscience™ (1:100) | Rat | 12-4801-82 | Invitrogen |
| CD3 Monoclonal Antibody (17A2), APC, eBioscience™ (1:200) | Armenian Hamster | 17-0032-82 | Invitrogen |
| CD4 Monoclonal Antibody (GK1.5), PE, eBioscience™ (1:200) | Rat | 12-0041-82 | Invitrogen |
| CD8a Monoclonal Antibody (53-6.7), eFluor™ 506, eBioscience™ (1:400) | Rat | 69-0081-82 | Invitrogen |
| CD25 Monoclonal Antibody (PC61.5), PE-Cyanine7, eBioscience™ (1:100) | Rat | 25-0251-81 | Invitrogen |
| Pacific Blue™ anti-mouse CD62L Antibody (1:200) | Rat | 104423 | BioLegend |
| APC/Cyanine7 anti-mouse/human CD44 Antibody (1:200) | Rat | 103028 | BioLegend |
| FOXP3 Monoclonal Antibody (FJK-16s), FITC, eBioscience™ (1:100) | Rat | 11-5773-82 | Invitrogen |
| CD71 (Transferrin Receptor) Monoclonal Antibody (R17217 (RI7 217.1.4)), FITC, eBioscience™ (1:200) | Rat | 11-0711-81 | Invitrogen |
| Alexa Fluor® 647 anti-mouse TER-119/Erythroid Cells Antibody (1:200) | Rat | 116218 | Biolegend |
| CD45R (B220) Monoclonal Antibody (RA3-6B2), Alexa Fluor™ 700, eBioscience™ (1:100) | Rat | 56-0452-82 | Invitrogen |
| CD93 (AA4.1) Monoclonal Antibody (AA4.1), PE, eBioscience™ | Rat | 12-5892-82 | Invitrogen |
| APC anti-mouse IgM Antibody (1:100) | Rat | 406509 | BioLegend |
| CD19 Monoclonal Antibody (eBio1D3 (1D3)), eFluor™ 506, eBioscience™ (1:100) | Rat | 69-0193-82 | Invitrogen |
| PE/Cyanine7 anti-mouse CD43 Antibody (1:400) | Rat | 143210 | BioLegend |
| CD24 Monoclonal Antibody (M1/69), FITC, eBioscience™ | Rat | 11-0242-82 | Invitrogen |
