# Supplementary material for: Hypoxia inducible factor 1α-driven steroidogenesis impacts systemic hematopoiesis
Source: Cell Mol Biol Lett. 2025 Aug 25;30:101. doi: 10.1186/s11658-025-00777-9 (PMC12379443; doi:10.1186/s11658-025-00777-9)
Supplement: Supplementary file 3 — Additional file 3. [file 11658_2025_777_MOESM3_ESM.pptx]

## Slide 1
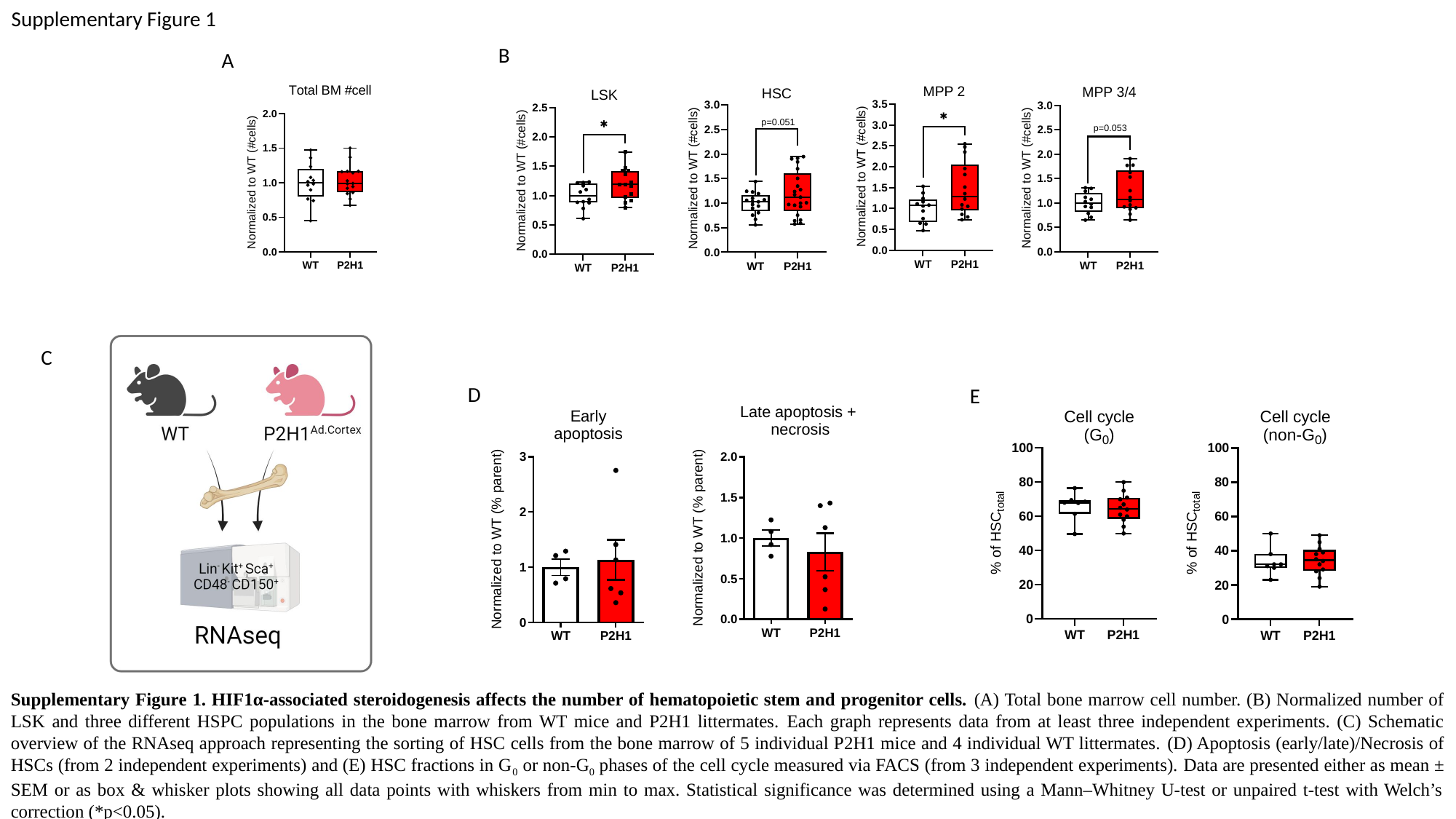

Supplementary Figure 1
B
A
C
D
E
Supplementary Figure 1. HIF1α-associated steroidogenesis affects the number of hematopoietic stem and progenitor cells. (A) Total bone marrow cell number. (B) Normalized number of LSK and three different HSPC populations in the bone marrow from WT mice and P2H1 littermates. Each graph represents data from at least three independent experiments. (C) Schematic overview of the RNAseq approach representing the sorting of HSC cells from the bone marrow of 5 individual P2H1 mice and 4 individual WT littermates. (D) Apoptosis (early/late)/Necrosis of HSCs (from 2 independent experiments) and (E) HSC fractions in G0 or non-G0 phases of the cell cycle measured via FACS (from 3 independent experiments). Data are presented either as mean ± SEM or as box & whisker plots showing all data points with whiskers from min to max. Statistical significance was determined using a Mann–Whitney U-test or unpaired t-test with Welch’s correction (*p<0.05).

## Slide 2
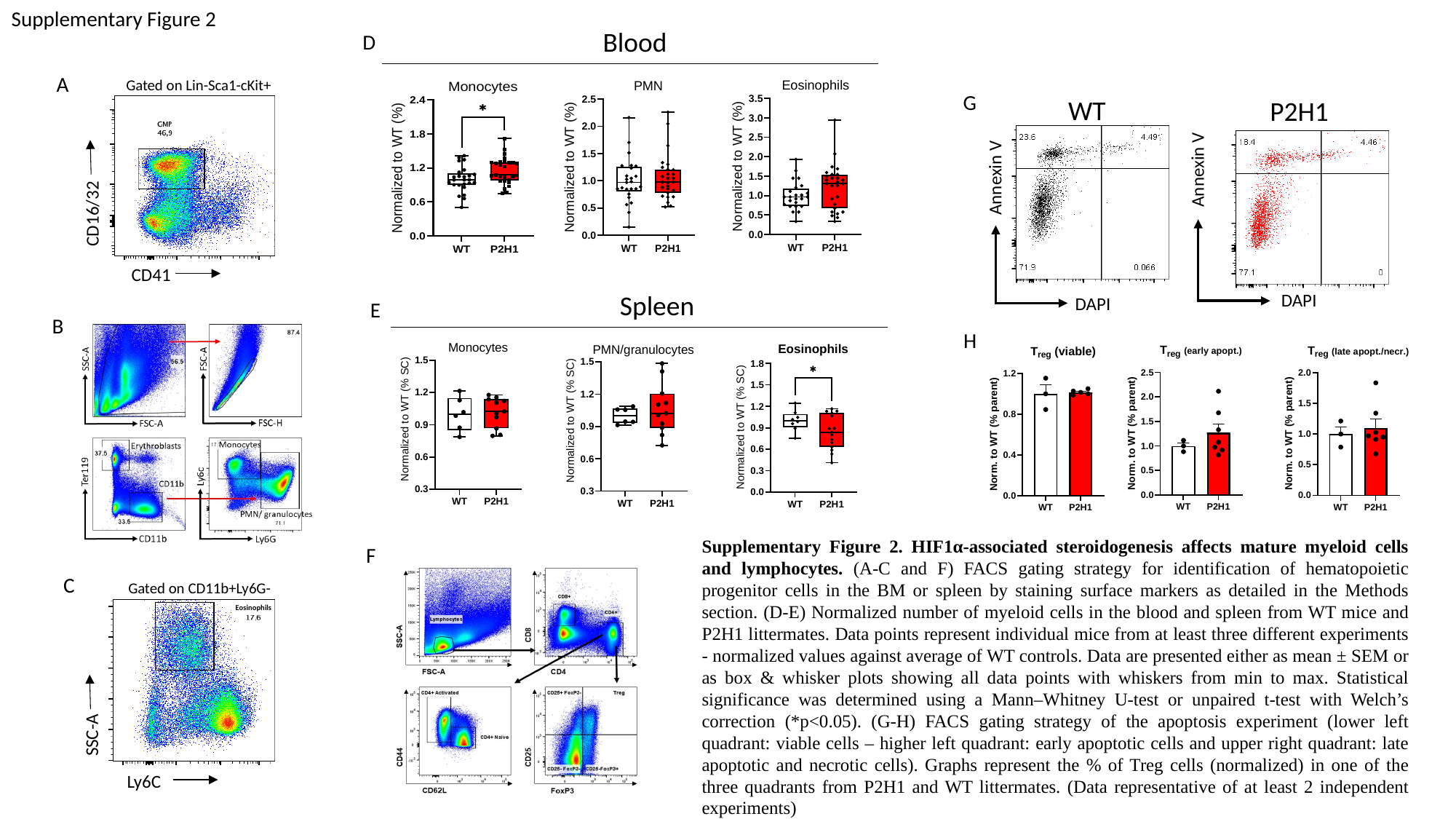

Supplementary Figure 2
Blood
D
A
Gated on Lin-Sca1-cKit+
G
WT
P2H1
Annexin V
Annexin V
DAPI
DAPI
CD16/32
CD41
Spleen
E
B
H
Supplementary Figure 2. HIF1α-associated steroidogenesis affects mature myeloid cells and lymphocytes. (A-C and F) FACS gating strategy for identification of hematopoietic progenitor cells in the BM or spleen by staining surface markers as detailed in the Methods section. (D-E) Normalized number of myeloid cells in the blood and spleen from WT mice and P2H1 littermates. Data points represent individual mice from at least three different experiments - normalized values against average of WT controls. Data are presented either as mean ± SEM or as box & whisker plots showing all data points with whiskers from min to max. Statistical significance was determined using a Mann–Whitney U-test or unpaired t-test with Welch’s correction (*p<0.05). (G-H) FACS gating strategy of the apoptosis experiment (lower left quadrant: viable cells – higher left quadrant: early apoptotic cells and upper right quadrant: late apoptotic and necrotic cells). Graphs represent the % of Treg cells (normalized) in one of the three quadrants from P2H1 and WT littermates. (Data representative of at least 2 independent experiments)
F
C
Gated on CD11b+Ly6G-
SSC-A
Ly6C
Eosinophils

## Slide 3
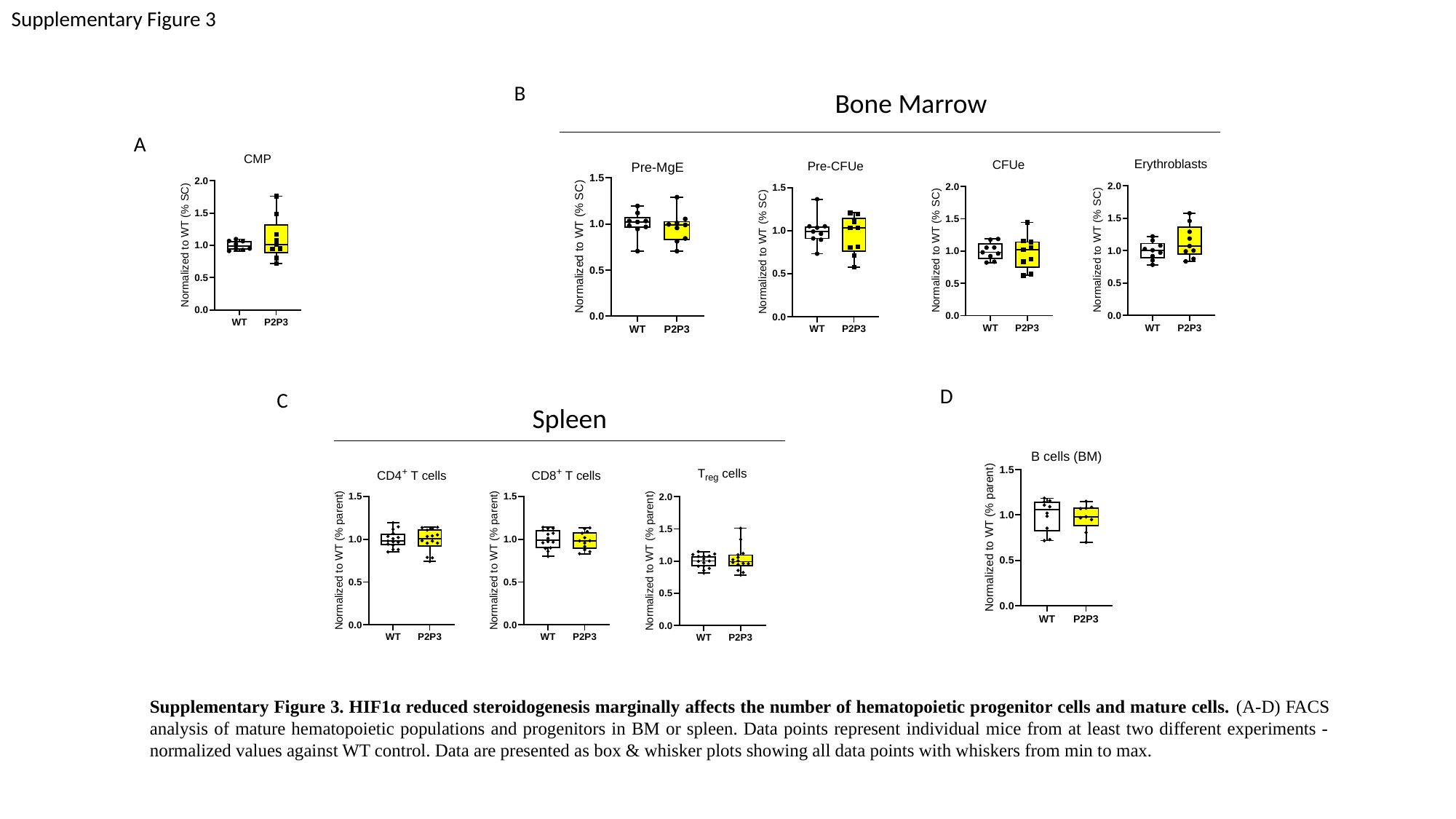

Supplementary Figure 3
B
Bone Marrow
A
D
C
Spleen
Supplementary Figure 3. HIF1α reduced steroidogenesis marginally affects the number of hematopoietic progenitor cells and mature cells. (A-D) FACS analysis of mature hematopoietic populations and progenitors in BM or spleen. Data points represent individual mice from at least two different experiments - normalized values against WT control. Data are presented as box & whisker plots showing all data points with whiskers from min to max.
